# Neuromagnetic Vistas into Typical and Atypical Development of Frontal Lobe Functions

**DOI:** 10.3389/fnhum.2014.00453

**Published:** 2014-06-18

**Authors:** Margot J. Taylor, Sam M. Doesburg, Elizabeth W. Pang

**Affiliations:** ^1^Department of Diagnostic Imaging, Hospital for Sick Children, Toronto, ON, Canada; ^2^Neuroscience and Mental Health Program, Hospital for Sick Children Research Institute, Toronto, ON, Canada; ^3^Department of Medical Imaging, University of Toronto, Toronto, ON, Canada; ^4^Department of Psychology, University of Toronto, Toronto, ON, Canada; ^5^Department of Paediatrics, University of Toronto, Toronto, ON, Canada; ^6^Division of Neurology, Hospital for Sick Children, Toronto, ON, Canada

**Keywords:** faces, inhibition, language, connectivity, ASD, preterm, developmental cognitive neuroscience, magnetoencephalography

## Abstract

The frontal lobes are involved in many higher-order cognitive functions such as social cognition executive functions and language and speech. These functions are complex and follow a prolonged developmental course from childhood through to early adulthood. Magnetoencephalography (MEG) is ideal for the study of development of these functions, due to its combination of temporal and spatial resolution which allows the determination of age-related changes in both neural timing and location. There are several challenges for MEG developmental studies: to design tasks appropriate to capture the neurodevelopmental trajectory of these cognitive functions, and to develop appropriate analysis strategies to capture various aspects of neuromagnetic frontal lobe activity. Here, we review our MEG research on social and executive functions, and speech in typically developing children and in two clinical groups – children with autism spectrum disorder and children born very preterm. The studies include facial emotional processing, inhibition, visual short-term memory, speech production, and resting-state networks. We present data from event-related analyses as well as on oscillations and connectivity analyses and review their contributions to understanding frontal lobe cognitive development. We also discuss the challenges of testing young children in the MEG and the development of age-appropriate technologies and paradigms.

Social function, executive processes, and speech are all complex cognitive processes that rely on the intact development and function of the frontal lobes. This paper will review, in turn, magnetoencephalography (MEG) studies addressing each of these three processes in typical development. Then, we will describe two clinical conditions – children with autism spectrum disorder (ASD) and children born very preterm – where these processes consistently, and differentially, follow an altered developmental trajectory. We will review our neuroimaging results that explored the involvement of the underlying neural bases of difficulties or dysfunction experienced in these domains. This contrast between typical and atypical development allows us a better understanding of which neural mechanisms are recruited, and at which stage of processing, to facilitate successful cognitive processing, and, it offers insight into how these mechanisms are compromised in clinical populations who experience difficulties in frontal lobe functions.

## Social Cognition

Social cognitive function refers to the capacity to adjust and manage successfully in social settings, which relies on executive abilities and on intact frontal lobe structure and function. Current models conceptualize executive processes as dependent on a network of frontal lobe regions with strong reciprocal connections to subcortical and parietal areas (Elliott, 2003). The frontal lobes are among the last brain regions to develop, and they play a critical role in executive abilities (Shaw et al., 2008). The maturation of social cognition parallels the development of the frontal lobes. Social cognitive functions have been strongly linked with medial prefrontal and anterior cingulate cortex (Bush et al., 2000; Radke et al., 2011; Telzer et al., 2011), which are inter-connected with dorsolateral and inferior frontal regions with connections to the superior temporal sulcus (STS) (Carter and Pelphrey, 2008; Kramer et al., 2010) and subcortical regions including the basal ganglia and amygdala (e.g., Satpute and Lieberman, 2006). This cognitive network is activated by a range of social and emotional tasks, including social judgment, facial affect, inhibition (Go/No-go), and empathy protocols. Disturbances of frontal lobe development can have severe consequences for the maturation of executive functions (Powell and Voeller, 2004). The present review focuses on the contribution of MEG for the study of frontal lobe function maturation and its relation to cognitive abilities, with a particular focus on executive functions and social cognition in both typically and atypically developing populations. We place specific emphasis on the development of emotional processing and inhibition, as well as the use of MEG to investigate network connectivity involving frontal lobe systems.

### Development of emotional face processing

The human face plays a vital role in human social interactions. Faces convey a tremendous amount of information, and the ability to differentiate and recognize faces/individuals and their emotional content has an extended developmental course through adulthood [see Kolb et al. (1992) for review]. Although posterior brain areas are crucial for face processing, frontal cortices play a critical role in deciphering the social significance of facial expressions and in allocating appropriate attention. Thus, emotional facial perception involves a distributed network that includes the amygdala, frontal lobes, anterior cingulate, STS, and fusiform gyri (McCarthy et al., 1999; Allison et al., 2000; Haxby et al., 2000; Adolphs et al., 2002; Kilts et al., 2003).

Numerous neuroimaging studies have investigated face processing in adults; however, knowledge regarding the developmental course of such abilities remains scant. Differences in frontal activation between adolescents and adults have been reported in fMRI in emotional regulation tasks (Burnett et al., 2009; Passarotti et al., 2009), as well as in tasks of emotional self-regulation and empathy (Lamm and Lewis, 2010). The timing of brain processing in the development of emotional face perception throughout childhood and adolescence has been determined with event-related potentials (ERPs), with the early emotion-specific responses emerging only in adolescence (Batty and Taylor, 2006; Miki et al., 2011). Due to its uniquely good combination of spatial and temporal resolution, MEG has been able to contribute tremendously to our understanding of the spatiotemporal dynamics underlying the processing of faces (Taylor et al., 2008, 2010, 2011a,b, 2012). With emotional faces in an explicit recognition task, early frontal activation that reflected implicit emotional processing was observed, whereas later insula and fusiform activity was found to be related to explicit emotional recognition (Bayle and Taylor, 2010).

Spatiotemporal dynamics of implicit brain processing of happy and fearful facial emotions has been established in adults using MEG (Hung et al., 2010). In this study, faces were presented rapidly and concurrently with a scrambled pattern, one on each side of a central fixation cross. Participants were instructed to respond to the scrambled image, thus not orienting attention to the face stimuli. This implicit emotional processing task revealed rapid activation of left amygdala at 100 ms for fearful compared with emotionally neutral faces. Increases in activity were observed concurrently in the dorsal ACC. The fast amygdala–ACC activation suggested a specialized frontal–limbic network, which may be responsible for facilitating responses to a potential threat. This study also confirmed that MEG source analyses could accurately measure both the location and time course of neurocognitive events in deep brain structures (e.g., Moses et al., 2007, 2009), as validated with simulated and real data analyses (Quraan et al., 2011; Mills et al., 2012).

This MEG protocol has also been used to investigate the development of neural activations associated with the implicit processing of fearful and happy facial emotions using school-aged children (7–10 years) and young adolescents (12–15 years) (Hung et al., 2012). Right lateralized amygdala activation to both happy and fearful faces was observed for the school-age children, whereas ACC activation did not reach statistical significance. In the young adolescent group, the pattern of neural activation similar to that observed in adults, left amygdala and ACC activation, was detected only during the perception of fearful faces. The results indicate that the processing of emotions first engaged the earlier-maturing amygdala, but was non-specific with respect to the emotion, and by the teenage years implicated the later-developing ACC. The maturational shift in the lateralization of amygdala responses sensitive to the fearful faces was intriguing, suggesting a shift from the more reflexive processing of the right amygdala to the elaborative processing typical of the left amygdala (e.g., Costafreda et al., 2008) with age. These results also inform our present views regarding the development of functional specialization of fear perception throughout childhood. Specifically, these findings indicate that this is a late-developing process involving the frontal–limbic emotion system, consistent with behavioral data on later maturation of recognition of negative emotions (De Sonneville et al., 2002; Herba and Phillips, 2004; Thomas et al., 2007).

### Executive functions: Inhibition abilities and imaging studies

Inhibition plays a vital role in social cognition, as inhibition of context-inappropriate behavior is critical for successful social functioning. Behavioral studies of inhibition indicate improvements in inhibitory control throughout childhood and adolescence (Luna et al., 2004). Presently, it is understood that inhibitory control is supported by a distributed network of brain areas, in which frontal cortex plays a pivotal role (Rubia et al., 2007).

Studies using fMRI in adults have identified brain regions underlying inhibition, which include striatal and thalamic structures, motor areas, anterior cingulate, parietal lobes, and the inferior and dorsolateral frontal gyri (Rubia et al., 2001; Watanabe et al., 2002; Mostofsky and Simmonds, 2008). Frontal cortex has been demonstrated to be critical for inhibitory control in studies that used Go/No-go tasks where comparisons were made between the activations during No-go (successful response inhibition) and Go trials (response execution) [see review in Dillon and Pizzagalli (2007)]. Brain imaging findings from Go/No-go paradigms in typical development have yielded variable results, yet have demonstrated a role for dorsolateral and inferior frontal regions in inhibition, although activation of this region is not reliably reported in children (e.g., Durston et al., 2002; Tamm et al., 2004; Rubia et al., 2007).

**Figure 1:**
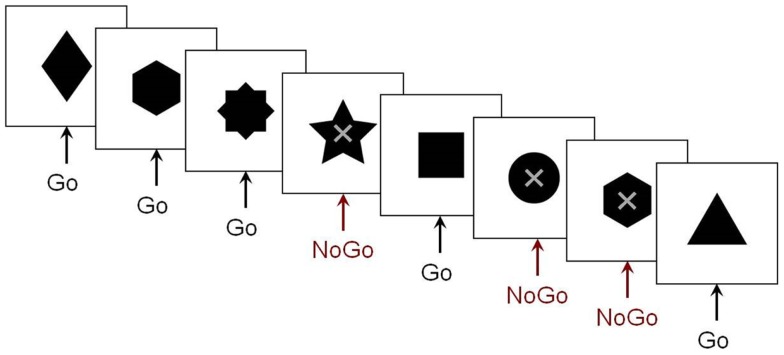
**The Go/No-go protocol**. Example of the stimulus sequence in the Go/No-go paradigm from our research group. Subjects were instructed to respond as quickly as possible to the variously shaped stimuli, but to withhold this pre-potent response on trials where an “X” was superimposed on the stimulus.

Fewer MEG studies of inhibitory control have been conducted. Our research group used MEG to investigate the maturation of spatiotemporal brain dynamics of inhibition in adolescence and early adulthood. We employed a visual Go/No-go task that included a baseline condition with many more No-go than Go trials, allowing us to compare the No-go trials between the two conditions, thereby avoiding contamination of MEG activity from motor responses (Vidal et al., 2012), seen in studies that contrast Go with No-go trials. The stimuli and sample series of stimuli from this paradigm are presented in Figure 1. Right-frontal activity beginning as early as 200 ms was observed in adults in the inhibition condition. Similar results were also reported in a prior ERP study (Bokura et al., 2001). Relative to adults, adolescents exhibited delayed frontal responses, which were also bilateral (Vidal et al., 2012). The low percentage (7%) of Go trials in the control condition, however, raised the prospect that the observed pattern of results may be attributable to an oddball effect. To address this potential confound, a follow-up study was run which included two variants of this paradigm: the frequencies of Go to No-go trials were reversed for the experimental (67:33%) and control (33:66%) conditions.

**Figure 2:**
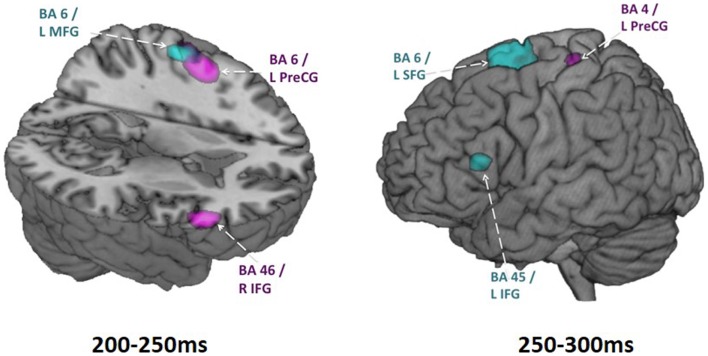
**Adolescent vs. adult inhibition activity**. Within group activations overlaid on brain images for time windows from 200 to 250 ms (on the left) and 250 to 300 ms (on the right). Inhibition condition > baseline condition, in adults is shown in magenta (*p* < 0.005, uncorr.) and in adolescents shown in blue (*p* < 0.005, uncorr.). Note particularly the earlier right IFG activation in adults and the later left IFG activation in adolescents. These analyses were conducted using vector beamforming on no-go trials that either did or did not (baseline) require inhibitory control. Beamforming was conducted on successive 50 ms windows from 200 to 400 ms. In this figure, the images from the baseline condition were subtracted from the inhibition condition for each group.

We examined spatiotemporal MEG dynamics in 15 adolescents and 15 adults using this more classic Go/No-go task. Comparison of brain activation during No-go trials using vector event-related beamformer revealed increased recruitment of right inferior frontal gyrus in adults (BA 45, at 200–250 ms), but bilateral and delayed activation of similar brain regions in adolescents (BA 45/9, at 250–300 ms) (Figure 2). Activation in the adolescent group was also observed in the right temporal (BA 21) and inferior parietal (BA 40) regions on inhibition trials. These additional activations may reflect increased recruitment of attentional processes (Durston et al., 2002; Hampshire et al., 2010). Delayed activation of frontal cortex, in concert with additional brain activation indicative of increased attentional demands (Vidal et al., 2012), indicate that brain networks mediating inhibitory control are not yet fully mature in adolescence.

### Language processing: Neural control of speech production in typical development

Speech production is a complex human behavior that requires the integration of articulatory control and oromotor control structures. Both non-speech mouth movements and speech-related mouth movements require the coordination of similar muscles and, presumably, similar neural pathways; however, the intentions behind the movements are different. Neuroimaging studies, primarily using fMRI, have compared the frontal cortical activation patterns during speech and non-speech oromotor tasks and report that the former was associated with more activation in left primary motor cortex, and the latter with bilateral and symmetric cortical activations (Wildgruber et al., 1996). It has been suggested that the motor cortex provides a common bilateral structural network for basic vocal motor tasks on which a left-lateralized functional network involved in the production of complex vocal behaviors, including speech and language production, is overlaid (Simonyan et al., 2009).

The spatiotemporal dynamics of speech production in humans can be studied with MEG. Thus far, MEG applications have been limited primarily to the study of language comprehension [for a review see Salmelin (2007)] as speech production generates high-magnitude artifacts that overwhelm the MEG signal. Various approaches have been used to address this challenge including the development of silent, covert, or imagined speech and language tasks (Dhond et al., 2001; Nishitani and Hari, 2002; Ihara et al., 2003; Vihla et al., 2006; Kato et al., 2007; Breier and Papanicolaou, 2008; Liljeström et al., 2009; Wheat et al., 2010; Pang et al., 2011). While silent tasks have addressed the significant artifact problem, they have unfortunately, also limited the study of the oromotor planning and motor control involved in speech and language production.

A few studies have examined overt speech and language production by using a subtraction method to remove the artifacts (Salmelin et al., 1994) or examining the neural responses prior to the onset of actual movements (Herdman et al., 2007; Carota et al., 2010). Another strategy has been to filter out high-frequency muscle-related activity and focus only on the 20 Hz beta-band motor responses to elucidate the neural regions involved in verbal and non-verbal lip, tongue (Salmelin et al., 1994), and mouth movements (Saarinen et al., 2006). As well, our group has used a spatial filtering approach to suppress artifacts from oromotor structures and have identified the sequence of neural activations involved in a simple oromotor task compared to a simple phoneme production task (Memarian et al., 2012). The successful application of this method in a control group allows us to use this approach in the examination of children with oromotor control and speech and language production difficulties.

## MEG Investigations of Frontal Lobe Functions in Autism Spectrum Disorder

Autism spectrum disorder is associated with abnormal social reciprocity, an intense desire for sameness, atypical use of language, and difficulties with speech. Difficulties with executive functions are prevalent in ASD, and poor social skills are present even when communication and cognitive abilities are high (Frith, 2004).

Numerous studies have identified atypicalities in brain development in ASD. How such alterations in neural development lead to symptoms and cognitive problems associated with ASD, however, remains poorly understood. Research from our group with children aged 6–14 years showed a trend for decreasing gray matter throughout childhood and early adolescence in typical children, but this pattern was not seen in children with ASD (Mak-Fan et al., 2012). Children with ASD also showed age-related atypical alterations in white matter, particularly in long-range fibers and areas linked to social cognition (Cheng et al., 2010; Shukla et al., 2011; Mak-Fan et al., 2013). The most reliably reported differences in brain volumes in ASD are in the frontal lobes, particularly in dorsolateral and medial frontal cortices (Carper and Courchesne, 2005), brain regions involved in social cognition (Lewis et al., 2011; Telzer et al., 2011). More recently, there has been tremendous interest in measures of functional and structural connections in the brain (e.g., Just et al., 2007, 2012; Müller et al., 2011; Travers et al., 2012; Schäfer et al., 2014), with studies finding that children with autism may have poorer long-range connectivity, which would negatively impact their ability to integrate information required for social interactions. Recent research from our group has demonstrated reduced MEG long-range theta-band coherence in children with ASD during the performance of an executive set-shifting task (Doesburg et al., 2013a). This reduced task-dependent synchronization included connections between frontal regions and a distributed network of brain regions, consistent with the view that poor executive ability in ASD may be linked with the inability of frontal structures to marshal coordinated activity among brain regions to support task performance.

### Social cognition deficits in ASD as assessed using emotional faces

The ability to perceive, recognize, and interpret emotional information in faces is critical for social interaction and communication. Impaired social interaction is considered to be a hallmark of ASD. Accordingly, studies designed to understand the bases for emotional face processing deficits in ASD play an important role in establishing the biological underpinnings of cognitive and social deficits in this group. Behavioral studies have consistently reported difficulties with face processing in children with ASD, and this population has also been shown to exhibit poor eye contact (Hobson and Lee, 1998) as well as a reduced tendency to look at the faces of others (Langdell, 1978). Prior work investigating neural responses to emotional faces in ASD have demonstrated activation of brain regions implicated in social cognition, including medial prefrontal and STS regions (Pierce et al., 2001; Pelphrey et al., 2007; Wang et al., 2007). ERP studies have also demonstrated that early responses to emotional faces in children with ASD were delayed and smaller (Wong et al., 2008; Batty et al., 2011). Such findings underscore the importance of MEG, as its uniquely good combination of spatial and temporal resolution permits accurate mapping of altered spatiotemporal dynamics in clinical child populations.

**Figure 3:**
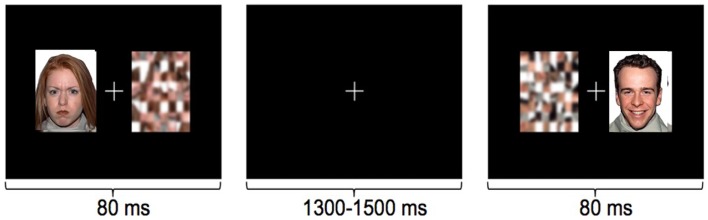
**Examples of the stimuli in the implicit emotional faces task**. Angry, happy, or neutral faces were presented to the left or right of fixation, with their matched scrambled faces. Participants responded with a left or right button press, as quickly as possible, to indicate the side of the scrambled pattern.

Using an implicit face processing paradigm in MEG (Figure 3), we investigated neural processing during the perception of happy and angry faces in adolescents with and without ASD. We chose angry instead of fearful faces, as this is a more commonly experienced emotion during childhood (Todd et al., 2012). In this study, emotional faces, adapted from the NimStim Face Stimulus Set (Tottenham et al., 2009), and scrambled images were presented concurrently on either side of a central fixation cross for 80 ms to adolescents with and without ASD; participants responded as quickly as possible indicating the side of the scrambled faces. Event-related beamforming analyses were performed on early MEG activation (60–200 ms). This revealed distinct brain activation patterns in response to happy and angry faces, which also differed between groups. We found significant group differences between the adolescents with and without ASD starting as early as 120–160 ms in source localized analyses, including greater left frontal activity in ASD, expressed particularly for angry faces.

**Figure 4:**
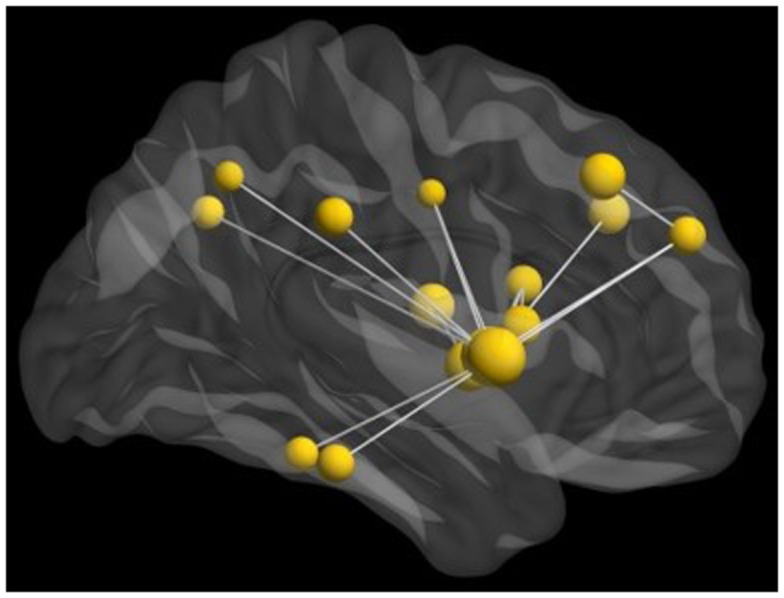
**Network of reduced beta-band connectivity seen in adolescents compared to matched controls during the implicit processing of angry faces**. The hub of this network was in the right insula, with connections notably to the fusiform gyrus and frontal lobes. For this analysis, data from seed regions were reconstructed using beamformer analysis. Data were then filtered into physiologically relevant frequency ranges, time series of instantaneous phase values were obtained using the Hilbert transform, and inter-regional phase locking was calculated using the phase lag index [PLI; see (Stam et al., 2007)].

We also examined connectivity with these data as inter-regional phase locking is a neurophysiological mechanism of communication among brain regions implicated in cognitive functions. When completed in task-based studies, these measures reflect the connectivity underlying task performance. Thus, we investigated inter-regional MEG phase synchronization during the perception of the emotional faces in this task. We found significant task-dependent increases in beta synchronization. However, beta-band inter-regional phase locking in the group of adolescents with ASD was reduced compared to controls during the presentation of angry faces. This decreased activity was seen in a distributed network involving the right fusiform gyrus and insula (Figure 4). This network also included activation in the right superior medial and dorsolateral frontal gyri; right fusiform and right supramarginal gyri; and right precuneus, left middle frontal, left insula, and left angular gyri.

Graph analysis of the connectivity in the beta-band, with the hub region of the right insula, revealed reduced task-dependent connectivity clustering, strength, and eigenvector centrality in adolescents with ASD compared to controls. Beta-band coherence has been suggested to be particularly important for long-range communication among brain regions, and these results suggest that reduced recruitment of this network, involving the limbic and frontal areas in the adolescents with ASD impacts their ability to integrate emotional information, particularly for angry faces.

In contrast, no significant differences were found in MEG responses to happy faces. These results are consistent with behavioral findings showing that high functioning ASD participants perform comparably to controls on tasks involving happy faces, but experience pronounced difficulties with processing angry faces (Kuusikko et al., 2009; Rump et al., 2009; Farran et al., 2011).

These findings are also in keeping with a broader literature reporting differences in functional connectivity between participants with and without ASD (e.g., Just et al., 2012; Khan et al., 2013) and suggest that these reductions in long-range task-dependent connectivity may be a factor in the social cognitive difficulties common in ASD.

### Difficulties with inhibitory control in ASD

Individuals with ASD often experience problems with inhibitory control, and this likely contributes to emotional outbursts and inappropriate social behavior commonly seen in this population. Inhibitory control has a protracted maturational course (Luna et al., 2010; Vidal et al., 2012). As such, investigation of these processes in adolescents with ASD is important, as this is a period when youths are adapting to increasing social demands. Moreover, it has been proposed that deficits in inhibitory control worsen with increasing age in ASD (Luna et al., 2004).

Several previous studies have investigated the neural underpinnings of inhibition difficulties ASD. For example in fMRI, adults with ASD have been shown to express greater left frontal activity (Schmitz et al., 2006), reduced anterior cingulate activity (Kana et al., 2007), together with atypical timing in the recruitment of frontal cortex. Since inhibitory control largely depends on the slowly maturing frontal lobes, the results from these studies in adults with ASD may not be generalizable to a younger population. Few neuroimaging investigations have been carried out during adolescence in ASD, when adult patterns of neural activity supporting inhibitory control are being established.

**Figure 5:**
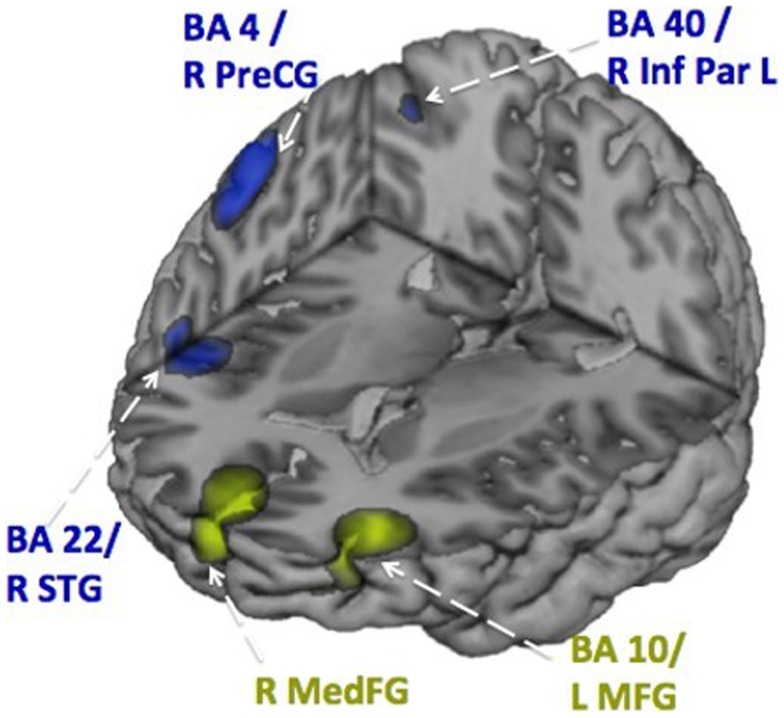
**Activation patterns during inhibition**. Distributed activation patterns at 350–400 ms in the adolescents with ASD (in green) and the age-matched typically developing adolescents (in blue) showing that control adolescents used a wider network, that also involved temporal lobe activation, than adolescents with ASD. These data were analyzed with vector beamforming as described in Figure 2.

To elucidate the maturation of inhibitory control in ASD, we recorded MEG while adolescents with ASD and age- and sex-matched controls performed a Go/No-go task (Vara et al., 2014) (see Figure 1). Participants were instructed to respond to Go stimuli and withhold responses to No-go stimuli (as described above). During inhibitory control, adolescents with ASD primarily recruited frontal regions, whereas typically developing controls showed bilateral frontal activation together with activation in temporal and parietal regions (Figure 5).

Results from this study suggest that inhibitory control is atypical in adolescents with ASD, whose false alarm rate was also higher than the control group, demonstrating poorer inhibitory control behaviorally. Also, patterns of the inhibition-related MEG activity in the adolescents with ASD exhibited different spatiotemporal neural processing than their matched controls. More extensive frontal activity was found in adolescents with ASD, which may be due to reduced long-range connectivity as well as increased short-range connectivity, or local over-connectivity (Belmonte et al., 2004).

### Atypical oromotor control and speech production in ASD

While ASD is characterized by deficits, most notably in the realm of social cognition, children with ASD have a variety of speech and language difficulties, the neurobiology of which is not well understood. We used MEG to examine a group of children with ASD as they completed several oromotor speech tasks to explore the neural regions implicated in the control and execution of these functions.

A group of children with ASD and a group of age- and sex-matched controls completed three increasingly complex oromotor and speech tasks in the MEG. These tasks included a simple motor task (open and close the mouth), a simple speech task (speak the phoneme/pa/), and a simple speech sequencing task (speak the phonemic sequence/pa-da-ka/). Beamforming analyses identified neural sources of interest which were then interrogated to generate the time courses of activation for the ASD and control groups. The peaks of activation were identified in each participant, and the latency and magnitude of prominent peaks were submitted to statistical testing.

**Figure 6:**
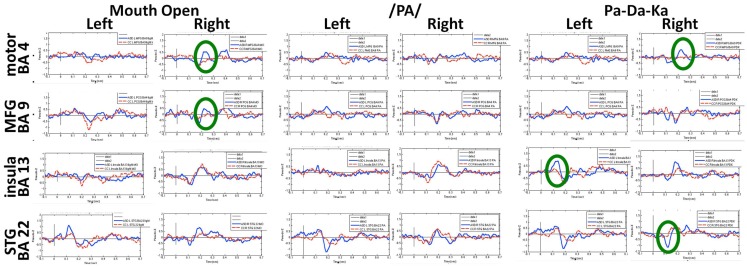
**Time courses of activations used in magnitude analyses for each speech production condition**. Circles indicate neural locations where statistically significant differences were seen in the peak magnitude between children with autism (blue line) and control children (red line). These data for Figures 6 and 7 were analyzed with synthetic aperture magnetometry beamforming (Robinson and Vrba, 1999; Vrba and Robinson, 2001), and peak activation in regions of interest (based on nine *a priori* regions identified from canonical expressive language areas, plus each homologous region) were computed and averaged by group, and then the magnitude (amplitudes) and latency analyzed for each participant and submitted to paired *t*-tests and corrected for multiple comparisons.

**Figure 7:**
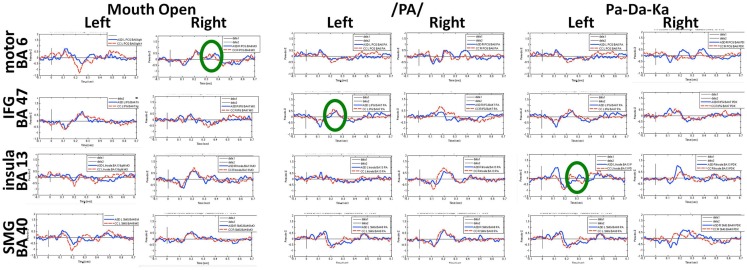
**Time courses of activations used in latency analyses for each speech production condition**. Circles indicate neural locations where statistically significant differences were seen in the peak latencies between children with autism (blue line) and control children (red line).

Figures 6 and 7 show the time courses where significant differences were observed in magnitude and latency, respectively, between the ASD and control groups, in three frontal areas and one temporal region (Pang et al., 2013). In the simple oromotor task, the children with ASD showed greater activation in right hemisphere motor control areas (BA 4) and the middle frontal gyrus, a motor planning area (BA 9), together with delayed activation in right hemisphere motor planning areas (BA 6). In the phoneme production task, the children with ASD showed delayed activation in left frontal language control areas (BA 47). In the sequencing task, the ASD group showed greater activation in right hemisphere motor control (BA 4) and right hemisphere sequencing (BA 22) areas. As well, for the sequencing task, the children with ASD showed higher and delayed activations in the left insula (BA 13), an area known to be involved in sensorimotor integration (Hesling et al., 2005).

These results fit with reports of difficulties with oromotor control and challenges with more complex speech patterns in children with ASD. In summary, we observed atypical neural activation in frontal areas associated with motor control, speech production, and speech sequencing. These were characterized by an unusual pattern of laterality (mostly in the right hemisphere), with higher magnitude and delayed activations. It is likely that these neurophysiological abnormalities underlie some of the speech/language difficulties observed in this cohort and may contribute to the speech and language deficits frequently experienced in children with ASD.

## MEG Studies of Frontal Lobe Development in Preterm Children

A large and growing number of children are now being born prematurely. Although the survival rate of these tiny infants has improved significantly due to advances in neonatal care, the morbidity rate has not changed and developmental difficulties are becoming increasingly noted (Roberts et al., 2010). Injuries to developing white matter systems are frequent in this group (Khwaja and Volpe, 2008) and even in the absence of injury on conventional MR imaging, atypical development of white matter has been reported (Anjari et al., 2007; Dudink et al., 2007). This has resulted in increasing interest in the relation between brain network connectivity and cognitive outcome in preterm children. The development of frontal lobe systems is of particular interest, as children born preterm often experience selective developmental difficulties with executive abilities, even when intelligence is broadly normal (Anderson Doyle, 2004; Marlow et al., 2007; Mulder et al., 2009).

Considerable investigation into relations between cognitive outcomes and atypical structural and functional brain development has been carried out using MRI [see Hart et al. (2008), Ment et al. (2009), Miller and Ferriero (2009)]. More recently, MEG has begun to emerge as a modality for imaging atypical development in preterm-born children. Early somatosensory responses have been shown to be atypical in preterm infants (Nevalainen et al., 2008), and these alterations are associated with illness severity (Rahkonen et al., 2013). Task-dependent MEG responses have been shown to be atypical in children and adolescents born prematurely and are associated with cognitive outcome (Frye et al., 2010; Doesburg et al., 2011a). MEG imaging had also been shown to be effective for revealing relations between specific aspects of adverse neonatal brain development, spontaneous brain activity, and school-age cognitive outcome in particular domains in preterm-born children (Doesburg et al., 2013b).

**Figure 8:**
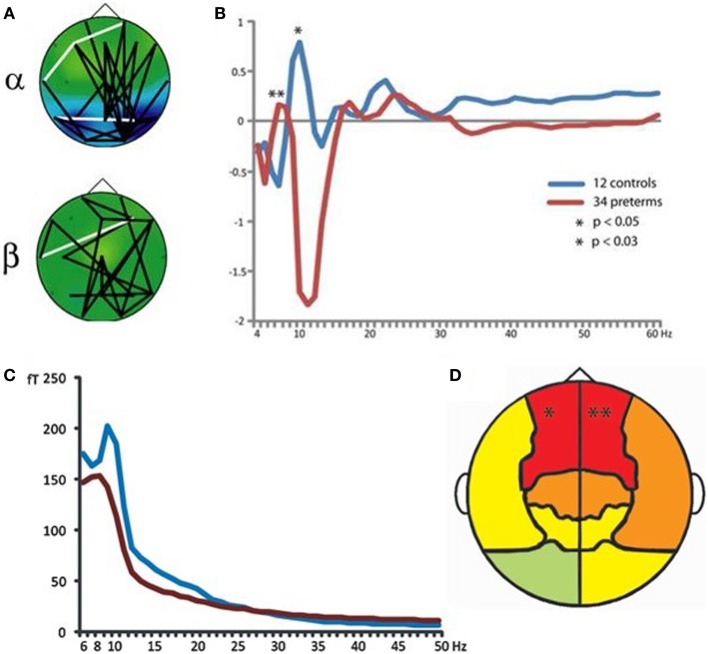
**Atypical neural oscillations in very preterm children**. **(A)** Increased long-range phase synchrony during short-term memory retention in typically developing controls. **(B)** Altered long-range synchronization during memory retention in very preterm children, suggesting that alpha-band connectivity may be slowed toward the theta frequency range. **(C)** Slowing of spontaneous MEG oscillations in very preterm children (blue line represents typically developing controls; red line represents preterm children). **(D)** Regional analysis of oscillatory slowing in very preterm children indicates involvement of frontal lobes. For these analyses, bandpass filtering and the Hilbert transform were used to obtain phase and amplitude values for each frequency and sensor. Long-range phase synchronization was indexed using phase locking values [PLVs; see (Lachaux et al., 1999)].

Magnetoencephalography has also been shown to be of specific relevance for understanding atypical development of frontal lobe systems in children born preterm. Frye et al. (2010) demonstrated altered frontal lobe activation during language processing in preterm-born adolescents, which was interpreted as compensatory top–down control from prefrontal cortical regions. Visual short-term and working memory retention has been demonstrated to recruit increased phase coherence between frontal and posterior brain regions in multiple frequency ranges, with alpha-band oscillations playing a pivotal role in task-dependent coupling (Palva et al., 2005, 2010a,b). This pattern of task-dependent network coherence is also robust in school-age children (Doesburg et al., 2010a; Figure 8A). In contrast, school-age children born very preterm exhibit reduced inter-regional coherence and atypical regional activation during visual short-term memory retention (Cepeda et al., 2007; Doesburg et al., 2010b, 2011a). This was manifest as reduced task-dependent inter-regional synchronization in preterm children, which was correlated with cognitive outcome in this group (Doesburg et al., 2011a). Close inspection of the spectral signature of task-dependent connectivity suggested that alpha coherence might be slowed in preterm children, as inter-hemispheric phase synchronization was significantly reduced at alpha frequencies, but increased in the theta range (Figure 8B).

Analysis of the resting-state MEG activity has also indicated that spontaneous alpha oscillations are slowed toward the theta range, and that this effect is maximal at sensors located over frontal cortex (Doesburg et al., 2011b; Figures 8C,D). Subsequent analysis of slowing of alpha oscillations in preterm children confirmed the involvement of prefrontal cortical regions (Doesburg et al., 2013c). Taken together with results from task-dependent network coherence, these findings suggest that atypical oscillatory activity in frontal lobe systems may lead to reduced ability to recruit network coherence supporting cognitive abilities. In this view, selective developmental difficulties with executive abilities prevalent in preterm-born children may be in part attributable to the inability of frontal lobe systems to recruit task-dependent inter-regional communication, mediated by neuronal synchronization. This perspective is supported by observations that alterations in both spontaneous and task-dependent alpha oscillations are associated with poor cognitive outcome in children born prematurely (Doesburg et al., 2011a, 2013b,c).

## Practical Considerations for Conducting Developmental MEG Studies

We have reviewed the contribution of MEG imaging for investigation of typical and atypical development in frontal lobe systems. Many of the studies reviewed demonstrate dramatic changes in the timing, location, and in connectivity, which continue throughout childhood, adolescence, and early adulthood, underscoring the utility of MEG for understanding the evolution of neural processes underlying cognitive abilities. Successful conduction of MEG imaging in children, however, requires several practical considerations, which we review here. The reader is referred to Pang (2011) for a detailed discussion.

The most common technical challenge to overcome when conducting MEG research with children is that of movement artifact. Although voluntary head and eye movements are reduced through training, researchers should be aware that tasks may need to be lengthened and trial numbers increased to allow for rejection of trials containing these artifacts. Moreover, MEG research in atypically developing child populations often requires a more liberal movement threshold, relative to what is typical for normative adult studies, in order to strike an appropriate balance between artifact-free recordings and an unbiased sample. New MEG systems with continuous head localization hold good promise for correcting for head movements, but these solutions may only be valid within a small range of movement and may not be able to correct appropriately for the movements of an agitated, hyperactive, or uncooperative child. Moreover, the impact of head motion correction techniques on more recently introduced analysis approaches has not yet been fully tested.

Magnetoencephalography helmets designed for recording from children improve issues with head movement, as there is less room for participants to move. These child-sized MEG systems also place the sensors much closer to the surface of the head, resulting in significant improvement to the signal to noise ratio. In an attempt to have the child feel less enclosed, however, these systems do not always have adequate coverage over the anterior aspect of the head for recording or analysis of frontal lobe activity. Also, institutions using these systems still require an adult MEG as the helmets of these pediatric systems would not accommodate pre-teen or teenaged participants.

In the studies described in this review from our research group, we have primarily relied on participant training to mitigate the influence of head movement on MEG recordings. The team members responsible for running the studies are trained to ensure that all participants understand the importance of staying still and spend considerable effort gaining and maintaining positive rapport with and cooperation from the children. Moreover, subjects are monitored throughout recording and reminded to remain as still as possible whenever necessary. Participants are also offered breaks as needed. Testing all children lying down, supine in the MEG, applying padding into the dewar to stabilize the head, as well as covering the child with a blanket to reduce body movement and thus head movement also facilitates cleaner recordings.

Task design is another critical consideration in developmental MEG studies. Experimental paradigms need to be age-appropriate, simple, understandable, engaging, and quick. One effective strategy is to first successfully implement an adult version of a protocol, usually adapted from a standard experimental neuropsychological test, and to subsequently pare the task down to its core features. Using colorful and child-friendly versions of these tasks is critical to maintain task performance long enough to obtain a sufficient number of MEG trials for subsequent reliable data analyses.

Taking these various considerations into account significantly reduces problems associated with MEG recordings in children, including clinical populations, allowing cognitive studies to be successfully completed. In our experience, the examination of cognitive functions using MEG is feasible in children as young as 4 years of age. For the youngest participants and clinical populations, the MEG is a much easier neuroimaging environment, as it is totally silent and less daunting than an MRI.

## Summary

We review MEG investigations of several frontal lobe functions associated with typical and atypical development – in social cognition, executive function, and speech – as well as network connectivity involving frontal lobe systems. This research highlights the complex and protracted developmental trajectory of frontal lobe systems, as well as the unique contribution that MEG can make to this field as a non-invasive neurophysiological imaging modality with a uniquely good combination of spatial and temporal resolution. We present some examples of atypical frontal lobe development, with relation to problems with cognitive development, in children with ASD and children born very preterm from a neuromagnetic imaging perspective. Finally, practical considerations for MEG imaging in clinical child populations and typically developing children are discussed. We believe that the range of applications briefly reviewed here highlight the promise of MEG for the comprehensive elucidation of typical and atypical development of frontal lobe processing and its relation to complex cognitive functions.

## Conflict of Interest Statement

The authors declare that the research was conducted in the absence of any commercial or financial relationships that could be construed as a potential conflict of interest.
